# Polyploid races, genetic structure and morphological features of earthworm *Aporrectodea rosea* (Savigny, 1826) (Oligochaeta, Lumbricidae) in Ukraine

**DOI:** 10.3897/compcytogen.v5i2.968

**Published:** 2011-06-01

**Authors:** R.P. Vlasenko, S.V. Mezhzherin, A.V. Garbar, Yu. Kotsuba

**Affiliations:** 1 Ivan Franko Zhytomyr State University, Velika Berdychevska 40, Zhytomyr 10008, Ukraine; 2 Dep. Evol. Genet. Basis of Systematics. Schmalhausen Institute of Zoology NAS of Ukraine, B. Khmelnytskogo 15, Kiev 01601, Ukraine

**Keywords:** earthworms, *Aporrectodea rosea*, genetic structure, polyploid races, morphology

## Abstract

Four chromosomal races (2n=36, 3n=54, 6n=108, 8n=144) and 96 clones have been revealed among 224 specimens of the earthworm *Aporrectodea rosea* over the territory of Ukraine by means of karyological analysis and biochemical genetic marking. Each population has been showed by several clones at least; moreover the clones from different places have never been identical. The clones in the range of one population can be identified with the set of quantitative and qualitative parameters.

## Introduction

Nowadays scientists are deeply interested with polyploid complexes of wide-spread animal species as most of such complexes became model objects for various experimental studies. Such interest has been caused by the conversion of the typological conception of species into the evolutional one. One of such complexes is the *Aporrectodea rosea* (Savigny, 1826) species complex that is widely spread in Europe and in particular over the territory of Ukraine.

Karyological studies (Muldal 1952, Omodeo 1952, Vedovini 1973, Mezhzherin et al. 2007, etc.) have showed the apomictic (parthenogenetic) species represented by series of heteroploid forms. These forms are considered to appear as a result of serial hybridization of several diploid species (Victorov 1993). As Vsevolodova-Perel and Bulatova (2008) reviewed, the populations of *Aporrectodea rosea* have turned out to have diploid (2n=36) or polyploid chromosome sets (3n=54, 4n=72, 4n=74–86, 5n=90, 6n=108, 8n=144, 10n=160–174).

Though the apomictic species may be represented by heteroploid forms, the one may contain from ten to several hundreds of clones (genotypes) with one ploidy level that can be revealed by means of analysis of allozyme variability (Lokki and Saura 1980; Jaenike et al. 1982; Terhivuo and Saura 2006).The origin of heteroploid forms may be connected with hybridization of different parental species, and forming of clones within one chromosomal level does with spontaneous mutation process. The hybrid nature and genetic mosaicism of apomictic forms stimulate great theoretical interest; though they reveal the problems in practical systematics as hybrids have intermediate morphology comparing to parental forms and mask the status of parental species. One should also consider the transgressive character of diagnostic features of differentiation of parental forms. As a result the analysis of morphological variability of genetically marked material parental species and hybrid forms appears to be necessary.

The structure of diploid-polyploid complex based on gene marking of *Aporrectodea rosea* was studied for several areas of this prevailing species only. There is only Fennoscandia in Europe, where the study of genetic structure of *Aporrectodea rosea* has been carried on (Terhivuo and Saura 1993, 1996, 2006). They have never carried on this earthworm over the territory of the Eastern Europe up to nowadays. Preliminary data from *Aporrectodea rosea* Ukrainian populations (Garbar and Vlasenko 2007, Mezhzherin et al. 2007, Vlasenko et al. 2007) have showed that this species is represented by several chromosome races with different ploidy and its high clone variability let it consider one of the most variable taxa of earthworms. Thus further studying of this species genetic structure on the territory of Ukraine by means of biochemical gene marking and cytogenetic methods appears to be actual.

## Materials and methods

**Figure 1.**
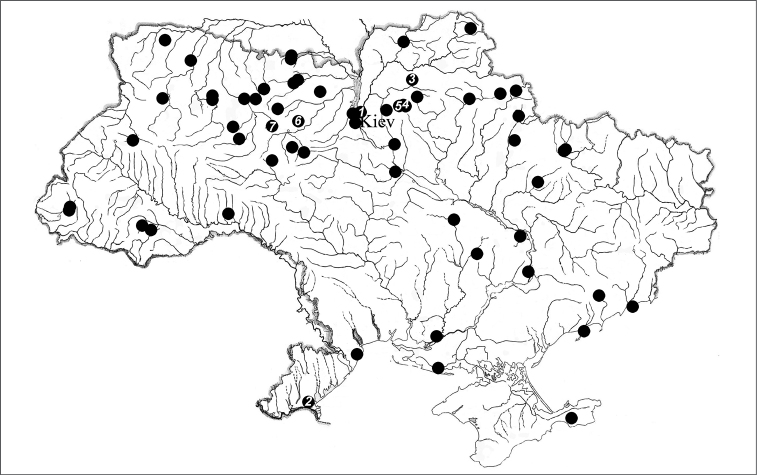
Localities of *Aporrectodea rosea* samples in Ukraine. Numbers are given to the most representative samples studied in the centre of country (**1** Kiyv, state capital) and in the south (**2** Vylkove; Odessa region), east (**3** Nizhyn, **4** Novy Bykiv, **5** Serbi; Chernigiv region), west (**6** Zhitomir, **7** Romanov; Zhitomir region).

The materials for the present study were collected in spring - autumn period of 2004 - 2008 using generally accepted procedure (Byzova et al. 1987). 224 specimens identified as *Aporrectodea rosea* according to the tables of Perel (1979) and Vsevolodova-Perel (1997) were used for karyological investigation. 60 samples from the Ukraine have been examined (Fig. 1). For the analyses, the most representative were samples from O.V. Fomin Botanical garden, Kyiv and Vylkove town, Odessa region, placed in the centre and south of the country (sites 1 and 2 in Fig. 1, resp.). Most often considered in the text are samples from the east and west to Kiyv containing specimens from sites 3–5 (Chernigiv region) and 6–7 (Zhitomir region).

Electrophoretic variability of spectra of enzymes (aspartate aminotransferase (AST), malate dehydrogenase (MDH), nonspeciﬁc esterases (ES) and superoxide dismutase (SOD) in extracts from a caudal part of body was investigated by the method of PAG–electrophoresis in Tris-EDTA-borate buffer system (Peacock et al. 1965).

Chromosome preparations were made from seminal vesicles following the technique formerly used by the authors in Lumbricidae investigations (Garbar and Vlasenko 2007). The earthworms were injected with 0.1% colchicine 24 hours before the dissection. The spermatocytes were placed for 50 minutes in distilled water and ﬁxed in the 1:3 mixture of glacial acetic acid and ethanol. Chromosome preparations were obtained by the reprint method (Sitnikova et al. 1990). Dried slides were stained with Giemsa-Romanovsky stain in 0.01M phosphate buffer (pH 6.8). The chromosome spreads were analysed with a «Mikmed» microscope (10×90).

The morphological studies have been started on the alive samples with defining the pigmentation of animal body and clitellum. The further studies have been held on the fixed earthworms when the body length (L) and clitellum (l1), the distance from the frontal part of the body to the clitellum (l2), the maximal diameter of the body out of the clitellum (d) have been measured. We have counted the quantity of segments (n1) and the quantity of segments up to the clitellum (n2), defined the head lobe form, the distance between the setae, the disposition of spinal pores and papilles, the size and position of clitellium, the form and position of pubertate platens by magnifying lens. Then the quantity of segments per 1 mm of body (n1/L), the quantity of segments per 1 mm of body up to the clitellum (n2/l2), the comparative thickness of the body (l2/d) have been measured. Statistical processing of obtained data was carried out by means of a package of applied statistical programs PAST 1.18.

## Results and discussion

### Biochemical gene marking

**Figure 2.**
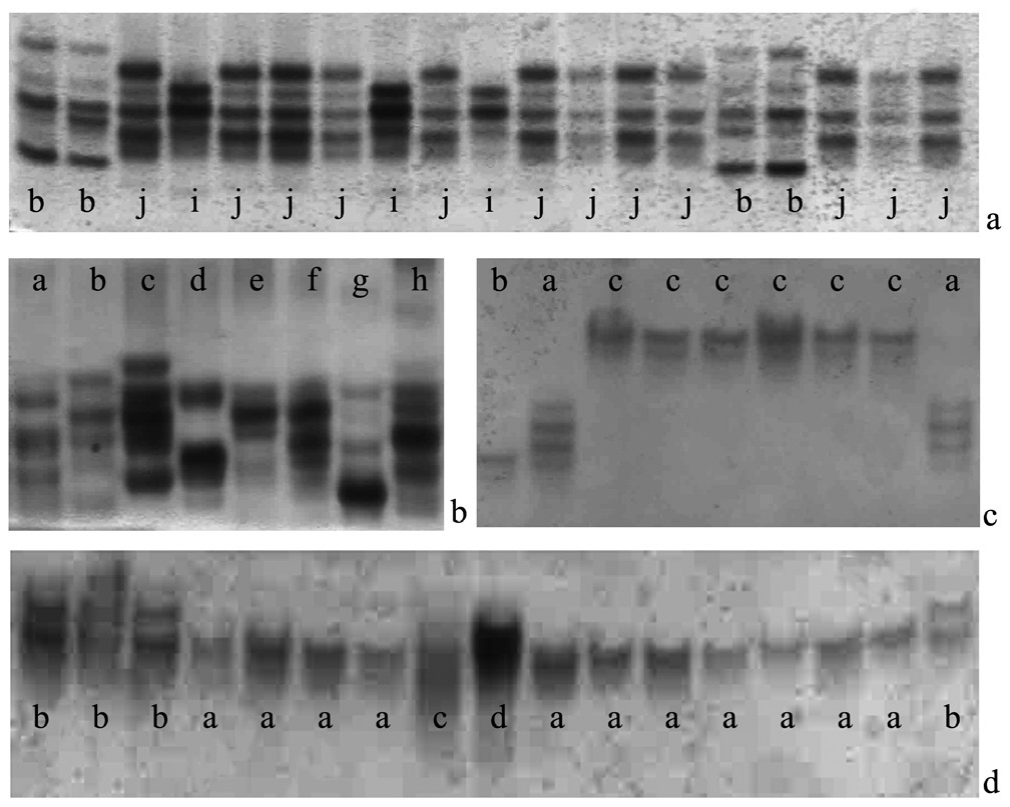
The geographic sample variability of *Aporrectodea rosea* electrophoretic enzyme spectra: nonspecific esterases (**A** Kiyv; **B** Novy Bykiv); aspartate aminotransferase (**C** Vylkove); malat dehydrogenase (**D** Serbi); different clones are signed by letters.

*Nonspecific esterases.* Nonspecific esterases of *Aporrectodea rosea* are coded by more than four loci. Despite of the very high variability level with the quantity of fractions in one spectrum varying greatly it is difficult to show the proper quantity of loci for even one organism. The analysis of variability of this enzyme’s spectrum proves the clone nature of variability by the fixation of the proper electrophoretic types. This way there the populations of reasonable variability (for example, the sample from O.V. Fomin National Botanical garden, Kyiv (Fig. 2a) with 16 electrophoretic spectra of nonspecific esterases have been found for 94 studied specimens), and populations with each specimen having its own type of the nonspecific esterases (cf. Novyi Bykiv and Galytsya) (Fig. 2b) have been found. We should point out at the fact that specimens with the identical spectra of nonspecific esterases have occured in one sample.

*Aspartate aminotransferase.* It was shown by series of alleles for this species. These series have single (homozygotic) or triple (constant heterozygotic) spectra typical for dimerous protein (Fig. 2c). In the latter case they may have the effect of the gene dose. There have been 6 alleles found. Despite of the lower variability (comparing to the esterases’ one) this enzyme and its coding loci have important diagnostic meaning for the clones’ definition. It’s worth pointing out the cases of finding the organisms with alternative alleles in one sample (Fig. 2c) as this fact reveals their independent origin and the absence of their alliances.

*Malate dehydrogenase.* This enzyme system is considered more conservative than aspartate aminotranspherase one. However sometimes it also shows the clone characteristics of the organism. Quite often heterozygotes have the asymmetric spectra (Fig. 2d) that points at the effect of the gene dose. There 5 alleles have been found.

### Clone structure

*The study of all over the territory of Ukraine.* The 96 clones distributed over 60 localities examined have been defined by means of biochemical gene marking of 224 earthworms that have been defined as *Aporrectodea rosea* due to their morphological features. This way more than half of them (67 clones) have been individual specimens that was considered to be normal for highly variable apomictic earthworms (Terhivuo and Saura 2006). The maximal quantity of organisms for one clone of 30 specimens has been found in season sample series in Botanical garden (Kyiv).

The average quantity of specimens for one clone has appeared to be only 2.33 ± 0.10 with the standard dispersion of σ=3.63 for all samples of *Aporrectodea rosea*. The correlation of the average quantity and dispersion of this species proves the tendency of the organisms’ random distribution as to the clones. Some tendency to the negative binominal distribution must be caused by the presence of unrandomising samples as the one from the botanical garden with its high number of organisms in one sample that’s led to the unproportionally high clone representation of in this area. The estimation of biodiversity basing the Shannon-Weaver formula has showed the sample average index of 1.85 for samples of 5 and more specimens and the one of 5.72 for all the studied earthworms to characterize the high biodiversity. This fact gives the grounds for considering this species as so-called hypervariable taxon (Cywinska and Hebert 2002) being usual for small invertebrate agamospecies.

As most of the studied samples of the territory of Ukraine have been not numerous, the further discussion has been based on two most representative samples from Kyiv (site 1) and Vylkove (site 2).

*The study of some settlements.* The 13 clones have been defined by means of biochemical gene marking of 90 specimens of the *Aporrectodea rosea* population of Kiyv of the spring-autumn period 2006 (Fig. 1, Table 1). It appears to be 6.9 ± 0.27 specimens per clone. Among them 6 (46%) clones have been shown to be individual specimens and 3 of them (23%) have had only two specimens. These way 4 dominant clones have got only 31% of their general quantity. Besides as to the quantity of organisms (78) they get up to 86.6% of the sample. The quantity of the found organisms varies from 5 (*Aporrectodea rosea*-C) to 30 (*Aporrectodea rosea*-B) in these clones’ measures. The distribution of these clones according to the quantity of specimens appears to correlate the distribution of rare events (Puasson distribution) that corresponds to stochastic laws. The close indexes of average (M=6.9) and standard dispersion (σ=7.78) point out at the same fact.

**Table 1.** Genetic structure of *Aporrectodea rosea* clones.

Kyiv					Vylkove				
Clone	ES	AST	MDH	n	Clone	ES	AST	MDH	n
A	a	b	c	29	A	a	b	a	13
B	b	a	a	30	C	b	c	a	9
B’	b’	a	b	2	A’’	a	b	b	4
C	i	a	a	5	A’	b	b	d	1
D	j	a’	a	14	A’’’	e	b	a	1
D’’	d	a’	a	2	A’’’’	e	e	?	1
E	d	b	a’	1	C’’	a	c	a	1
F	e	c	b	1	C’’’	e	c	a	1
G	f	c	b	1	E	c	a	a	1
H	e	d	a	1	D’	d	b	a	1
I	c	a	a	2	F	t	b	a	1
K	h	a	a	1	B	c	d	c	1
L	i	e	c	1	D	a	a	?	1

**Figure 3.**
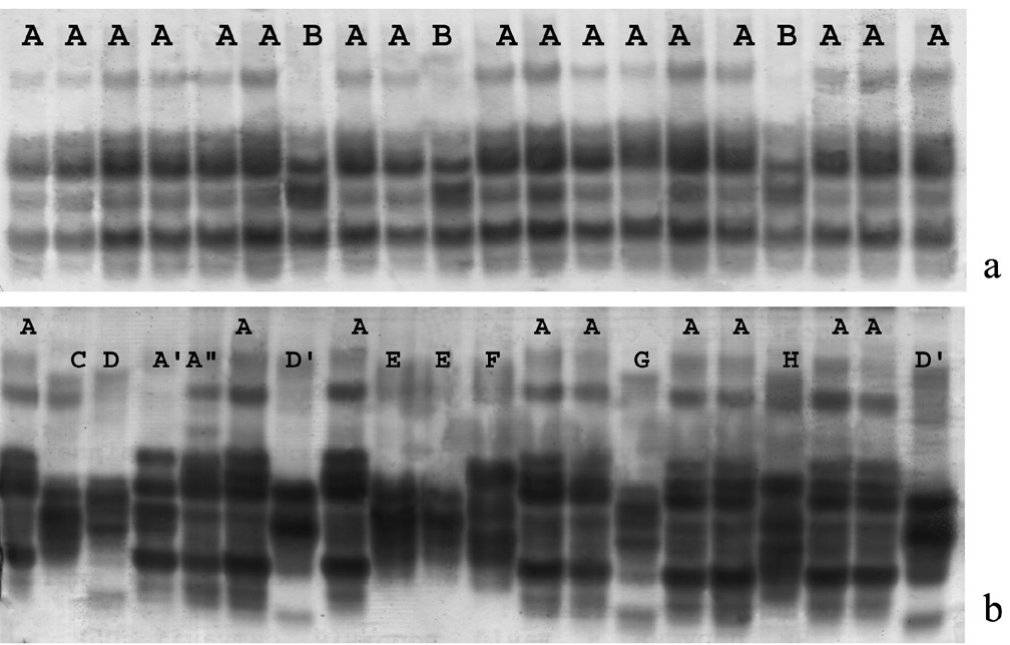
The seasonal variability of nonspecific esterases and clonal diversity of the *Aporrectodea rosea*  populations from the Kiyv sample: **a** June **b** October.

We have been able to observe the sudden changes in the clones structure of the different season samples (Table 2). This way it has been not only the rare clone structure that changes but the dominant one does as well (Fig. 3).

**Table 2.** The representation of *Aporrectodea rosea* clones in the season samples from Kiyv.

Period of the specimens collecting	clones	n
June-July 2005	A, B, C, D’	9
August 2005	A	10
May-June 2006	A, B, D’	13
July 2006	A, B, I, J, K	29
September 2006	B, B’, C, D	25
October 2006	D	3

The 13 clones have been defined by means of biochemical gene marking of 36 specimens of the *Aporrectodea rosea* population of Vylkove of the spring-autumn period of 2006 (Fig. 1, Table 1). It appeares to be 2.8 per clone. Among them 10 (76.9%) clones have been shown to be individual specimens and 3 dominant clones get 23.1% of their general quantity. Besides as to the quantity of organisms (26) they get up to 72.2% of the sample. The quantity of the found organisms varies from 4 (*Aporrectodea rosea*-C) to 13 (*Aporrectodea rosea*-B) in these clones’ measures. The distribution of these clones according to the quantity of specimens appears to correlate the Puasson distribution. The close indexes of average (2.76) and standard dispersion (3.83) point out at the same fact.

**Figure 4.**
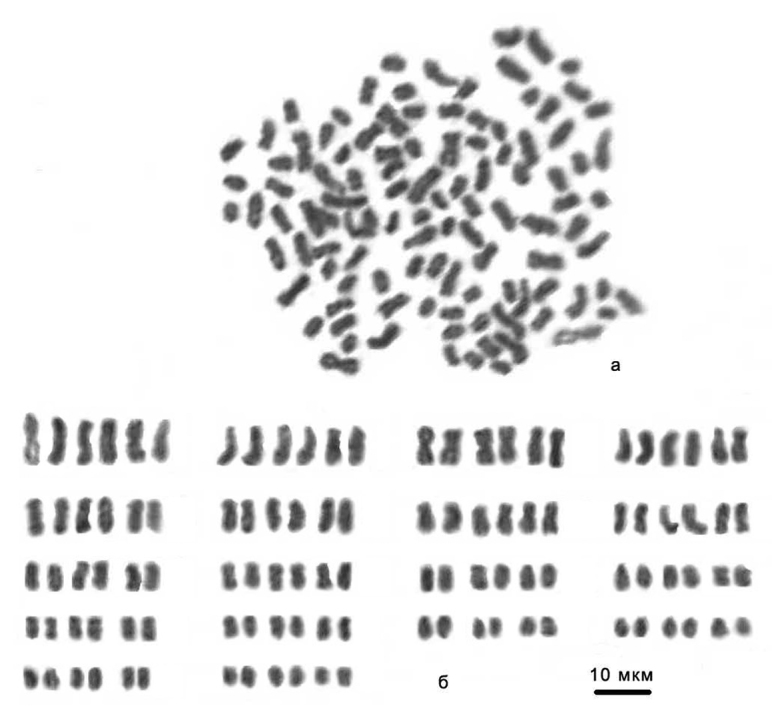
The hexaploid chromosome set (6n=108) of the clone *Aporrectodea rosea*-C from Kiyv: **A** mitotic metaphase **B** karyogram.

*Karyological analysis.* The specimens that could be used for karyological analysis have been obtained of only five *Aporrectodea rosea* samples which, however, represent 4 different regions in the centre, east, west and south of Ukraine (Table 3). In the Kiyv sample, 33 mitotic metaphases of *Aporrectodea rosea*11 specimens were studied. We have also showed the *Aporrectodea rosea*-A clone being characterized by triploid number of chromosomes 3n=34, NF=108 by analyzing five specimens. We have got 6 metaphase plates of two specimens of *Aporrectodea rosea*-B clone with diploid number of chromososmes (2n=36, NF=72). This clone must be represented by diploid race. The clone *Aporrectodea rosea*-C has been characterized by hexaploid number of chromosomes 6n=108, NF=216 (Fig. 4). The specimens of *Aporrectodea rosea*-D clone has been characterized by octaploid number of chromosomes (8n=144). The chromosomes of the triploid race have been typically smaller than the ones of the diploid race.

In the Vylkove sample, we have got 20 mitotic metaphases of the five specimens of the most spread clone A. The organisms of this clone have been found to have triploid number of chromosomes:3n=54, NF=108.

We have showed the triploid number of chromosomes 3n=54, NF=108 for samples from other regions, too. Eight specimens of Nizhyn sample, six specimens from Zhitomir and four from Romanov all were triploid and, above, one organism with hexaploid chromosome number have been found.

Thus, the series of heteroploid *Aporrectodea rosea* clones from different places of Ukraine were found by means of karyological analysis (Table 3). With all this going on dominant number of chromosomes is shown to be triploid considered its being the most spread over the territory of Ukraine (Viktorov 1993).

**Table 3.** Karyotypes of*Aporrectodea rosea* from Ukraine and their belonging to clones.

Sample (site on the map)	Clone	Karyotype	The quantity of plates	The quantity of specimens
Kiyv (1)	A	3n = 54	23	5
	B	2n = 36	6	2
	C	6n = 108	8	3
	D	8n = 144	4	2
Vylkove (2)	A	3n = 54	20	5
Nizhyn (3)	A	3n = 54	23	8
Zhitomir (6)	A	3n = 54	12	6
Romanov (7)	A	3n = 54	17	4
	B	6n = 108	2	1

*The analysis of morphological features of different clones representatives.* We have used the wide-spread*Aporrectodea rosea* clones of earthworms from Kyiv and Vylkove for morphological analyses. We should point out that the analysis of different clones of the same residence let’s avoiding the environmental effect that anyway leads to modificating the morphological appearance of earthworms in samples from different regions.

*Kiyv.*Average values of investigated parameters of four *Aporrectodea rosea* wide-spread clones (A, B, C, D) are given in Table 4. As for the results of dispersive analysis (LSD–test), *Aporrectodea rosea*-A clones (basing the absolute (D) and comparative (l2/D) body diameter) and *Aporrectodea rosea*-B clones (basing comparative body diameter) are well-differentiated ones for the morphological features, while no reliable difference between *Aporrectodea rosea*-C and *Aporrectodea rosea*-D clones have been found. The qualitative parameters also allow the reliable differentiating of most of clones (Table 5).

**Table 4.** Average (M) and standard errors (m) of morphological parameters of *Aporrectodea rosea* clones. L - length of whole body, l1- clitellum, l2- forward end of body, D - maximal diameter of a body behind clittelum, n1 - total number of segments, n2 - number of segments up to clitellum.

Parameters		Clone /Number of specimens						
		Kiyv				Vylkove		
		A(29)	B(30)	C(5)	D(14)	A(13)	C(4)	A’’(9)
L, MM	M	27,14	31	31,6	29,36	30,62	30,5	29,67
	m	1,26	0,88	1,12	1,09	1,49	1,04	2,86
n1	M	96,14	110,03	110,2	104,86	109,23	107,25	100,44
	m	3,52	2,39	3,32	2,39	4,95	5,3	10,02
n1/L	M	3,6	3,58	3,49	3,61	2,88	3,13	2,56
	m	0,06	0,05	0,02	0,08	0,23	0,13	0,23
n2/l2	M	2,86	2,85	2,87	2,98	7,5	7,25	7,72
	m	0,03	0,08	0,08	0,05	0,27	0,25	0,25
l2, mm	M	8,19	8,85	8,4	8,43	3,06	2,88	2,64
	m	0,05	0,32	0,24	0,14	0,13	0,13	0,13
l1, mm	M	3,28	3,09	3	2,93	2,47	2,53	2,96
	m	0,09	0,13	0,32	0,22	0,07	0,12	0,13
D, mm	M	2,15	1,92	1,7	1,71	3,6	3,53	3,38
	m	0,06	0,04	0,12	0,07	0,14	0,21	0,05
l2 /D	M	3,89	4,48	5	4,97	3,3	3,41	3,09
	m	0,06	0,11	0,2	0,11	0,12	0,1	0,11

**Table 5.** Frequencies (%) of qualitative parameters of*Aporrectodea rosea*.

Features	Variantsof parameters	Kiyv				Vylkove		
		A	B	C	D	A	C	A’’
		n= 29	n= 30	n= 5	n= 14	N=13	N=4	N=9
Beginningof clitellum(segments)	24	0	0	0	0	15.4	0	22.2
	25	100	100	100	0	15.4	25	77.8
	26	0	0	0	100	69.2	75	0
Endingof clitellum(segments)	31	3.5	0	0	0	7.7	0	0
	32	96.6	100	100	100	15.4	100	100
	33	0	0	0	0	76.9	0	0
Pigmentationof body	Unpigmented	0	0	0	0	100	100	66.7
	Light pink	100	100	100	100	0	0	33.3
Pigmentationof clitellum	Deep orange	96.6	0	0	100	100	0	0
	Light orange	0	100	0	0	0	0	22.2
	Pink and white	0	0	0	0	0	100	55.6
	Unpigmented	0	0	100	0	0	0	22.2
	Red	3.5	0	0	0	0	0	0

The discriminant analysis of the features’ parameters has showed the studied clones to be discriminated better within the qualitative features but for the quantitative ones (97.44 and 61.54% respectively). Considering all the features even leads to some lowering of the discrimination accuracy (96.15%) comparing to the one based on the qualitative parameters.

**Figure 5.**
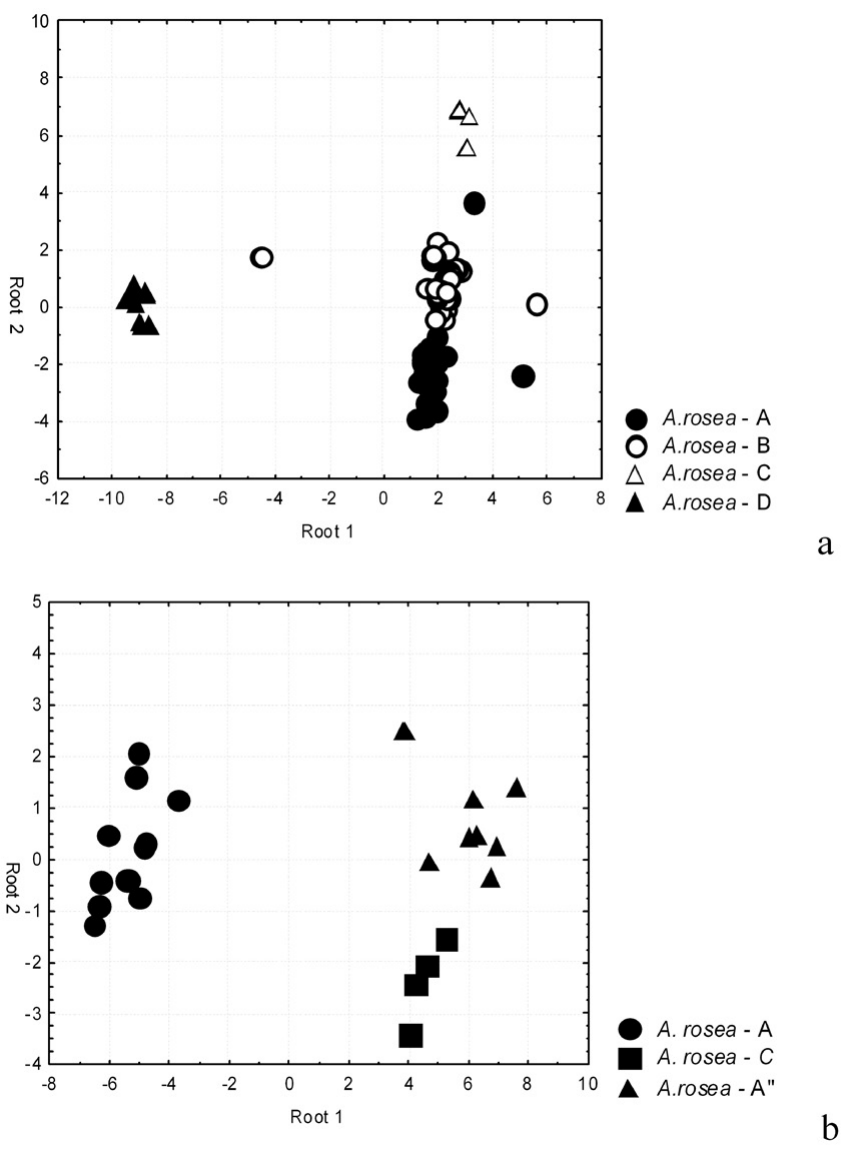
The distribution of the studied*Aporrectodea rosea* samples in the zone of discriminant functions: **a** Kiyv (north) **b** Vylkove (south).

The dispersion diagram (Fig. 5, a) shows only *Aporrectodea rosea*-D standing out for the first discriminant function. *Aporrectodea rosea*-A, *Aporrectodea rosea*-Band *Aporrectodea rosea*-C are differed with the second discriminant function. The using of other pairs of discriminant functions has given the same results.

*Vylkove.*We have used three most wide-spread clones(A, A’’, C) for morphological analysis.Average values of their investigated parameters are given in Table 4. The dispersive analysis (LSD–test) has showed the presence of their possible distinctions with some features. For example the *Aporrectodea rosea*-A *– A. rosea-*A’’ clone pair differ significally with the absolute (D) and relative (l2/ D) diameter of the body, though *Aporrectodea rosea*-C *- A. rosea*- A’’ does only with the relative one. Besides, the clones used for the analysis are reliably differentiated within the number of qualitative features (Table 5).

The discriminant analysis has showed all the studied clones to be discriminated well (100%) basing the set of features. The clone *Aporrectodea rosea*-A stands out well with the first discriminant function (Fig. 5, b). *Aporrectodea rosea*-A’’ and *Aporrectodea rosea*-C are differed with the second discriminant function. Thus all three dominant clones are districted from each other rather well.

## Conclusions

The incredibly high variability of *Aporrectodea rosea* clones has been found out (96 clones per 224 studied specimens from 60 localities) in our study. As a rule, several clones have been studied in the range of one population and the clones of different places have never been identical ones. We have expected for such results basing the Fennoscandian data (Terhivuo and Saura 2006). Authors have managed to find 46 clones per 155 studied organisms that comes to 3.4 organisms per one clone. Here the high interclonal and geographical variability of morphological features has become apparent. According to our data the clone variety of this species is higher for the territory of Ukraine than for Fennoscandia and comes to 2.33 organisms per clone.

Such a high level of genetic variability of this species must be induced by the high genome mutability that must be caused the recombination of the genetic material that has led to forming of not only lot of genetic form but the heteroploid races as well. For example di- (2n=36), tri- (3n=54), hexa- (6n=108) and octaploid (8n=144) *Aporrectodea rosea* specimens have been found on the studied territory. This way the triploids appear to be the most wide-spread ones and get nearly 73% of the general quantity of the studied specimens. The significant heterogeneity of this species distinctly correlates the high clone variability. We have managed to define the set of features for the clones’ identification in the range of one population. They are districted typically better with their qualitative features than with the linear parameters ones. Besides, the clone structure of*Aporrectodea rosea* population is characterized by distinct season variability that proves the ecological differentiation of clones. Further sampling is needed, however, to support the preliminary observations presented here.
